# Expression of Selected Integrins and Selectins in Bullous Pemphigoid

**DOI:** 10.1155/2007/31051

**Published:** 2007-04-02

**Authors:** Agnieszka Żebrowska, Anna Sysa-Jędrzejowska, Małgorzata Wągrowska-Danilewicz, Ewa Joss-Wichman, Anna Erkiert-Polguj, Elżbieta Waszczykowska

**Affiliations:** ^1^Department of Dermatology and Venereology, Medical University of Lodz, 94-017 Lodz, Poland; ^2^Laboratory of Nephropathology, Medical University of Lodz, 94-017 Lodz, Poland; ^3^Laboratory of Immunodermatology, Department of Dermatology and Venereology, Medical University of Lodz, 94-017 Lodz, Poland

## Abstract

Blister development in bullous pemphigoid (BP) results from destruction of hemidesmosomes and basement membrane components within the dermoepidermal junction by autoantibodies. Adhesion molecules can take part in pathogenesis of this disease. The aim of the study was to determine the localization and expression of L- and E-selectins and *β*1, *β*3, and *β*4 integrins by immunohistochemistry in skin lesions of 21 patients with BP, compared with 10 healthy subjects. Expression of L and E selectins and *β*1, *β*3 integrins was detected mainly in basal keratinocytes and in inflammatory infiltrates in the dermis, expression of *β*4 integrin was irregular and was detected mainly in dermal part of the blister, while in the control group only weak and single expression of the examined molecules was detected in basal keratinocytes and endothelium cells. The obtained results reveal the important role of selected selectins and integrins in development of skin lesions in BP.

## 1. INTRODUCTION

Development of skin lesions in the course of bullous pemphigoid (BP) results from destruction of basement membrane components. Complex structure of this membrane is responsible for maintaining the integrity of dermo-epidermal junction. Two glycoproteins of molecular mass 230 kD (BPAG1) and 180 kD (BPAG2) are the autoantigens in bullous pemphigoid. Structural studies revealed that extracellular fragment of BPAG2 with COOH-terminal collagenous domain connects the basement membrane with epidermal hemidesmosomes. NC16a fragment of BPAG2, located within its extracellular fragment, is thought to be the most immunogenic part of the antigen. Binding of autoantibodies directed against these autoantigens, localized in the basement membrane of the epidermis, activates a series of immunological and enzymatic phenomena [1, 2].

Inflammatory infiltrates in the dermis, formed by neutrophils and eosinophils, and bound in vivo IgG and C3 deposits along the basement membrane of the epidermis are observed. Ultrastructural studies confirmed also the presence of intensive inflammatory infiltrate at dermo-epidermal junction, as well as destruction of hemidesmosomes and components of extracellular matrix [3]. Formation of the infiltrates is preceded by early accumulation of leukocytes, depending on activity of adhesion molecules, especially selectins and integrins. Schmidt et al. [4] observed that binding of autoantibodies with BPAG2 leads to activation of keratinocytes, which release interleukin 6 and interleukin 8, as well as activation of C5 component of the complement. Mast cells and neutrophils activated by the same immunological reaction migrate through walls of blood vessels with help of E and L selectins and secrete specific proteases that can digest a series of basement membrane proteins. Matrix metalloproteinases, released by inflammatory cells and keratinocytes, are finally responsible for blister formation [5, 6]. Migration of immunocompetent cells from blood vessels into foci of inflammation has several phases. In each stage of this process different families of adhesion molecules are involved, responsible for rolling, adhesion, activation, binding, and diapedesis of leukocytes [7].

During the first stage (leukocyte rolling), binding of E and L selectins with oligosaccharide ligands results in the so-called marginalization of the cells, for example, direct contact of leukocytes with the endothelium. Second stage, leukocyte activation, is characterized by increased affinity of integrin receptors on rolling leukocytes. Cytokines produced locally in foci of inflammation cause halting of rolling cells and their pushing through the epithelium [6–8]. Subfamilies of integrins, for example, *β*1 and *β*3, are involved in the third stage, termed strong adhesion. During the fourth stage diapedesis and migration in tissues can be observed [6, 7, 9].

Adhesion molecules have an important role during further extravascular stages of migration. They are transmembrane glycoproteins composed of extracellular, intramembrane, and cytoplasmatic functional domains [7]. The *β*4 integrin is additionally involved in connecting the filaments of the cell to the basement membrane and forming hemidesmosomes. Destruction of these hemidesmosomes is an essential process in development of skin lesions in BP [3].

Continued research concerning modulation of activity of selected selectins and integrins that are involved in processes taking place within the skin may contribute to development of new therapeutic methods for various dermatoses, especially bullous skin diseases.

There are few literature data concerning variations of selected adhesion molecules serum levels or examinations on animal models in bullous pemphigoid. The aim of the study was to determine the localization and expression of E and L selectins and *β*1, *β*3, and *β*4 integrins in lesional skin of patients with bullous pemphigoid and in skin of healthy persons.

## 2. MATERIAL AND METHODS

The study included 21 patients with BP (15 women and 6 men, aged between 58 and 84 years, mean age 68.5) in active stage of the disease. All patients had skin lesions-blisters, vesicles, papules, or erythema. Control group consisted of 10 healthy persons (5 women and 5 men, ages between 19 and 49 years, mean age 42). The study was approved by local ethics committee of Medical University of Lodz.

Diagnosis of bullous pemphigoid was based on clinical picture as well as histological and immunological examinations. 11 patients had skin lesions in form of blisters, vesicles, and itching erythematous papules, the rest of patients presented with only erythema.

In all patients, direct immunofluorescence test revealed bound in vivo IgG/C3 linear deposits along basement membrane zone. In salt split test, the deposits were observed in epidermal part of the blister, or both in epidermal and dermal parts. By indirect immunofluorescence test circulating IgG antibodies were found in 17 out of 21 patients, while ELISA test (MBL, Nagoya, Japan) showed the presence of anti-NC16a autoantibodies in 19 cases. In histological examination, features consistent with diagnosis of BP were observed, such as neutrophilic, eosinophilic, and lymphocytic infiltrates, and in 11 cases—subepidermal blisters.

Immunohistochemistry was used to evaluate the expressions of studied adhesins. Biopsies for immunohistochemical examination were obtained from lesional skin in patients and from the nape region in healthy controls.

Paraffin-embedded sections were used for routine H + E staining and for immunohistochemistry in DAKO EnVision detection system using immunoperoxidase method. The following primary monoclonal antibodies were used: CD29 (*β*1 family), CD61 (GPIII) (*β*3 family), CD104 (*β*4 family), CD62E (E-selectin), and CD62L (L-selectin), (Novocastra, United Kingdom).

For immunohistochemistry the paraffin-embedded sections were placed on adhesive plates and dried at 56°C for 24 hours, later deparaffinated in a series of xylens and alcohols with decreasing concentrations (96%, 80%, 70%, 60%). Activity of endogenous peroxidase was then inhibited with 3% hydrogen peroxide solution in methanol for 5 minutes.

In order to retrieve the antigenicity of tissues and allow them to react with antibodies, the following procedures were used for each of the tested antibodies: for CD62E the sections were heated in 0.001 M versenian buffer (EDTA) of 8.0 pH in water bath at 95° for 30 minutes; for CD62L the sections were boiled in 0.001 M versenian buffer (EDTA) of 8.0 pH in microwave oven at 700 W for 15 minutes; for CD29 the sections were heated 6 times in 0.01 M citrate buffer of 6.0 pH in microwave oven at the following power levels: 150 W (5 minutes), 350 W (5 minutes), 450 W (5 minutes), and 650 W (6 minutes); for CD61 and CD104 the sections were heated in DAKO target retrieval solution in water bath at 95° for 30 minutes. Cooled sections were then rinsed in 0.05 M TRIS buffer (TBS) at 7.6 pH for 30 minutes and incubated for 60 minutes at room temperature in damp chamber with, respectively, diluted antibodies CD62E (E-selectin) 1 : 50, CD62L (L-selectin) 1 : 50, CD29 1 : 40, CD61 (GPIII) 1 : 25, CD104 1 : 50. After incubation the sections were rinsed twice in TBS buffer and DAKO EnVision double-step visualization system was next applied in order to visualize the antigen-antibody reaction.

The following semiquantitative scale was applied for evaluation of the intensity of immunohistochemical reaction of CD29, CD61, CD104, CD62E, and CD62L in skin biopsies: −: no reaction, +: weak intensity in most cells, 2+: moderate intensity, 3+: strong intensity.

Expression of selectins and integrins was assessed by two independent pathologists using Olympus BX 41 microscope (Japan). Whole slides with all fields of microscopic view were investigated.

## 3. RESULTS

Results of semiquantitative evaluation of expression of studied integrins and selectins in immunohistochemical examination are presented in Table 1.

### 3.1. Integrin *β*1 (CD29)

**Figure 1:**
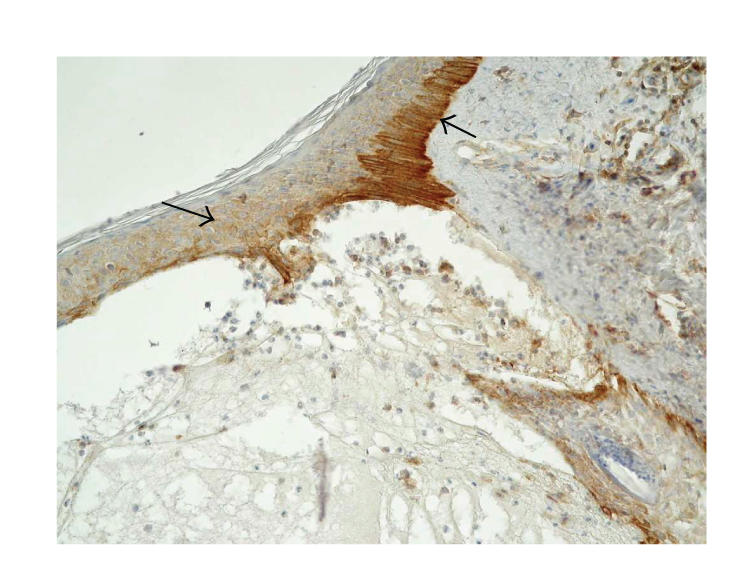
Skin lesions in BP, immunohistochemistry. Moderate expression of *β*1 integrin in basal keratinocytes and weak in the whole epidermis and blister fluid (original magnification x100).

**Figure 2:**
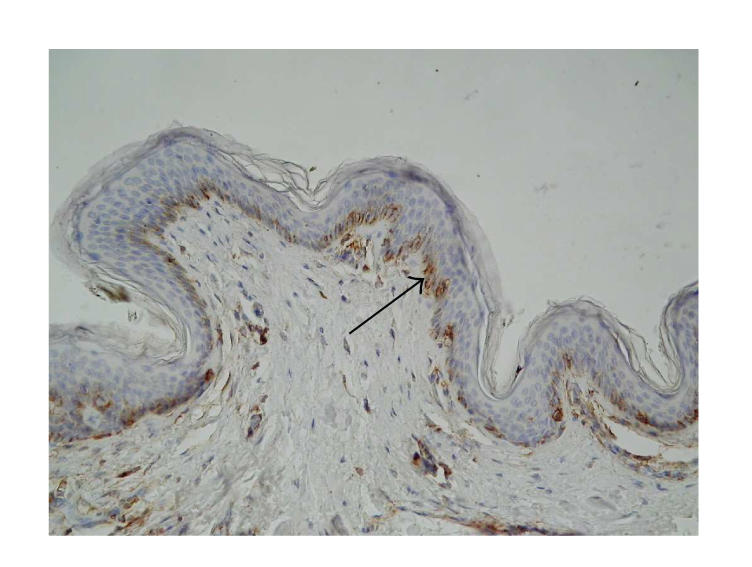
Healthy skin, immunohistochemistry. Signal for *β*1 integrin in single keratinocyte (original magnification x400).

Immunoexpression of integrin *β*1 (CD29) was detected in all basal keratinocytes. In skin samples from patients with BP, mean intensity of immunoexpression was weak (14/21) (see Figure 1). Moderate expression of CD29 was observed in 4 of 21 patients. In skin samples obtained from healthy volunteers expression was very weak and detected only in single cells (see Figure 2).

### 3.2. Integrin *β*3 (CD61)

**Figure 3:**
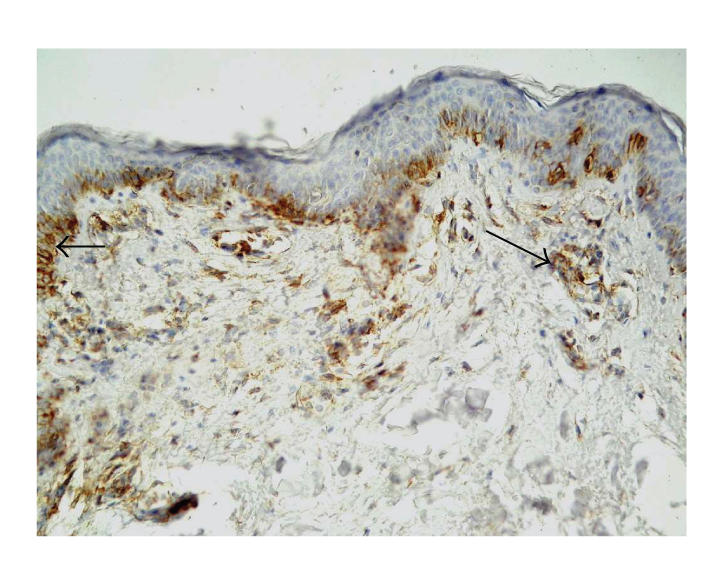
Skin lesions in BP, immunohistochemistry. Moderate expression of *β*3 integrin in basal keratinocytes and weak in the whole stroma (original magnification x100).

Reaction with CD61 antibody was positive in skin samples from patients and was localized in basal keratinocytes and focally also in other layers of the epidermis (see Figure 3). Mean intensity of immunoexpression of CD61 was weak (14/21). In control skin samples, immunoexpression of *β*3 integrins (CD61) was not observed.

### 3.3. Integrin *β*4 (CD104)

**Figure 4:**
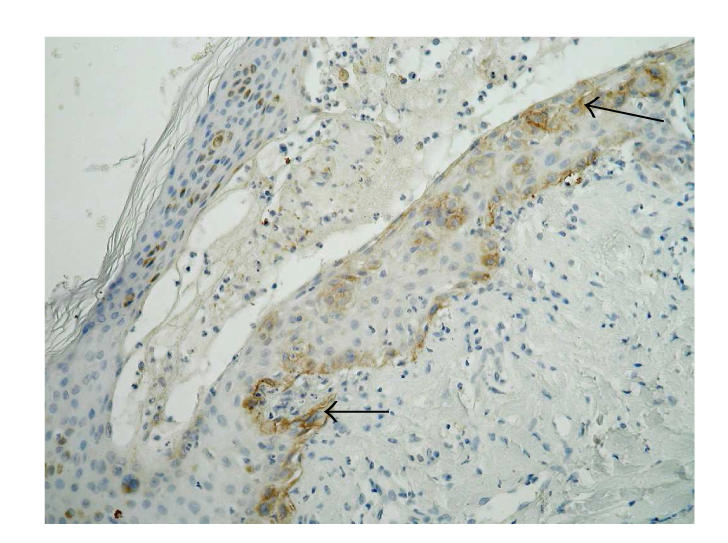
Skin lesions in BP, immunohistochemistry. Moderate expression of *β*4 integrin in the dermal part of blister and in blister fluid (original magnification x100).

**Figure 5:**
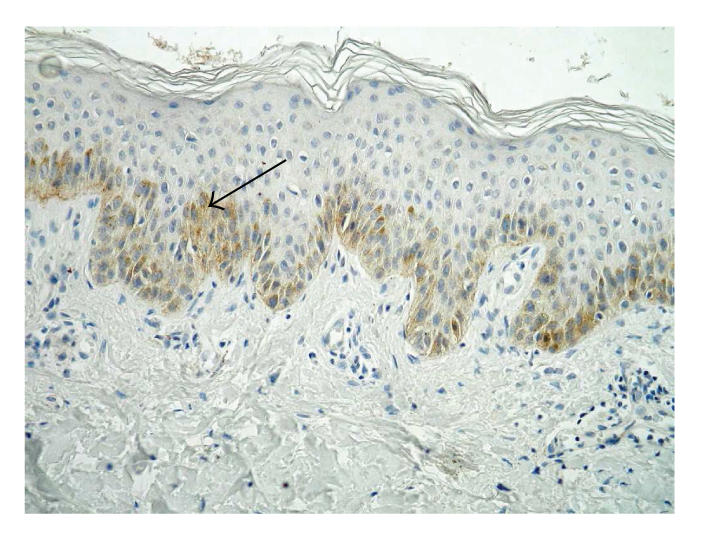
Healthy skin, immunohistochemistry. Signal for *β*4 integrin in single basal keratinocyte (original magnification x100).

Immunoexpression of integrin *β*4 (CD104) was detected in hemidesmosomes in BP samples as well as in control biopsies. In lesional skin biopsies from patients, expression was irregularly scattered along basement membrane (see Figure 4), while in healthy skin it was regular and linear (see Figure 5). Mean intensity of CD104 expression in pemphigoid was weak (14/21).

### 3.4. E-selectin (CD62E)

**Figure 6:**
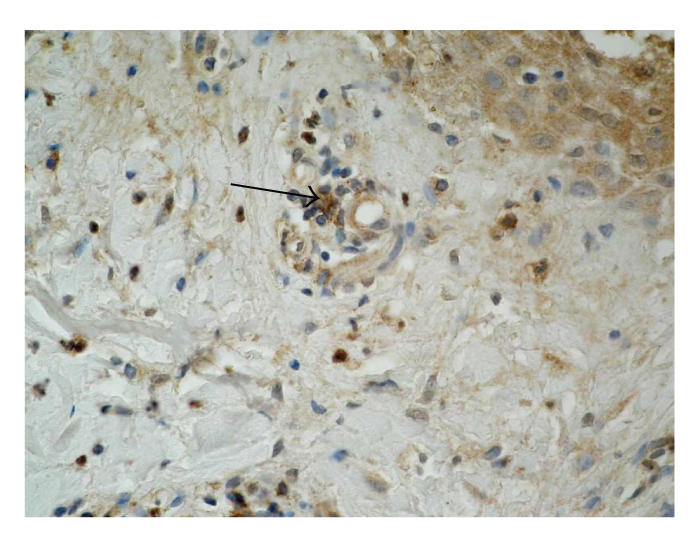
Skin lesions in BP, immunohistochemistry. Moderate expression of L-selectin on leucocytes (original magnification x100).

Immunoexpression of E-selectin in skin samples from BP patients was detected on endothelial cells and neutrophils (see Figure 6). Mean intensity of CD62E immunoexpression in patients was weak (17/21). In biopsies from healthy skin, only single endothelial cells showed very weak expression of CD62E.

### 3.5. L-selectin (CD62L)

**Figure 7:**
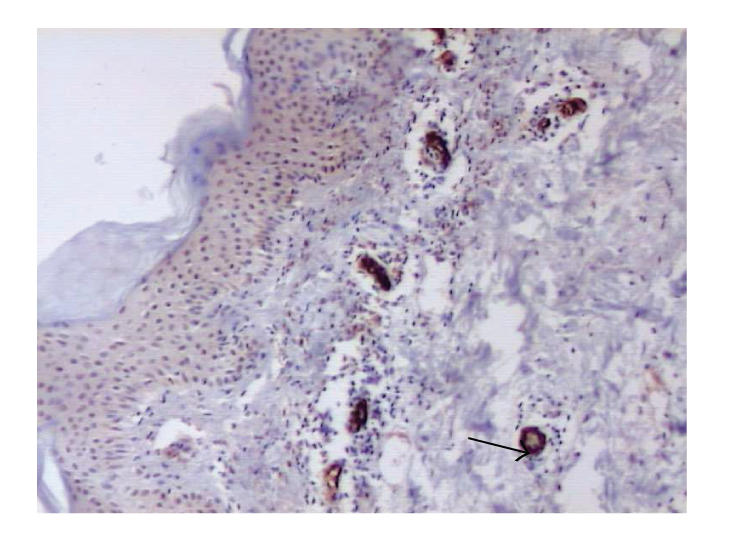
Skin lesions in BP, immunohistochemistry. Strong expression of E-selectin on endothelial cells (original magnification x100).

Immunoexpression of L-selectin was detected on limphocytes, macrophages, and neutrophils (see Figure 7). Mean intensity of expression of CD62L in pemphigoid was weak. In biopsies from healthy skin very weak expression was observed only on single endothelial cells.

## 4. DISCUSSION

Pemphigoid is a subepidermal bullous dermatosis in which pathological processes result in disconnection between basal layer of the epidermis and the dermis, causing formation of tense blisters. It was proved that administration of rabbit anti-mBP180 antibodies to newborn mice causes production of pathological antibodies, accumulation of leukocytes, and formation of complement deposits along basement membrane that results in development of blisters [4, 9]. Scare literature data revealed the role of certain adhesive molecules in the pathogenesis of BP. Recent studies established biochemical properties of metalloproteinases and their tissue inhibitors and their high affinity to components of the basement membrane zone. These enzymes are produced by eosinophils and neutrophils attracted to the basement membrane by selectins and integrins.

Integrins belong to particles that are very important for maintaining the dermo-epidermal junction as well as cell-to-cell connections. Lack of *β*1 integrin during fetal development in mice is a lethal mutation [8, 10, 11]. Genetic or autoimmune dysfunction of *α*6*β*4 integrin causes bullous lesions on the skin and mucous membranes in the course of such diseases as junctional type of bullous epidermolysis or cicatricans pemphigoid. Expression of various integrins on keratinocytes, firoblasts, endothelial cells, and migrating cells is also necessary for normal healing processes and for occurrence of the apoptosis phenomenon [12, 13]. Increased expression of integrins is associated with promotion of cell migration, production of metalloproteinases, and angiogenesis [14, 15].

Activation of various signal transduction pathways depends on the family of integrins, type of associated ECM protein, and stimulating factors. Integrin stimulating factors are cytokines, growth factors, chemokines, and other adhesion molecules, including cytokines themselves [14–16]. Some literature date confirm the role of these cytokines, chemokines, and ECM enzymes in pathogenesis of BP [4, 5].

Integrins are heterodimers build from *α* and *β* chains connected noncovalently that pass through the cytoplasmatic membrane, joining extra and intracellular environments [7, 17, 18]. Linkage of an integrin with ligand initiates signaling cascade that modulates cell behavior and gene transcription [19]. Integrins are main cellular receptors for binding with extracellular matrix components (e.g., plectin, fibronectin, vitronectin, collagen) through special short protein fragments and are involved in intercellular adhesion in epidermis [20, 21]. *β*1 subfamily integrins are involved mainly in the interactions between cells and connective tissue macromolecules (e.g., fibronectin, laminin, collagen); *β*2 are associated with cell-to-cell interactions, while *β*3 play a role in connections with ligands such as fibrinogen, vitronectin, thrombospondin, and von Willebrand factor [7, 18]. *β*1 integrin subunit was detected in upper and lateral parts of cellular membrane of basal keratinocytes, while it was very seldom observed in its lower parts. This observation may suggest that this molecule is involved mainly in maintaining the intercellular connection [20]. Molecules that belong to *β*1 subfamily of integrins may become expressed as late as 2 to 7 weeks after lymphocyte stimulation.

In our study, expression of *β*1 integrin in all basal keratinocytes in lesional skin biopsies from pemphigoid patients was observed, in comparison with very weak signal from only single cells in the control group, revealing the important role of lymphocyte stimulation and production of cytokines in this disease. The expression in all basal keraqtinocytes is an answer for significantly lymphocyte infiltration.

*β*3 integrin is a transmembrane glycoprotein, expressed mainly on platelets and megakaryocytes. It binds with CD51 molecule, creating a receptor for vitronectin, and thus becomes expressed in the cells of many tissues. Moreno et al. [11] used monoclonal antibodies against this integrin, revealing its presence in epithelium of mouse's kidneys and testicles. Structural and functional analysis of *β*3 integrin fragments showed its strong adhesive potential and ability to influence the function of fibrinogen, explaining the expression of this integrin during the inflammatory process as well as its presence on other cells, including epithelial cells [4, 22].

In our study, expression of this integrin was observed on single epidermal cells. Positive signal was detected in lesional skin biopsies, comprising basal keratinocytes or focally cells from other layers of the epidermis. It seems that the inflammatory process and destruction of dermo-epidermal junction may be induced by expression of *β*3 on keratinocytes, but such phenomenon should be confirmed by other diagnostic methods.

Hemidesmosomes are multiprotein complexes responsible for adhesion of epithelial cells to extracellular matrix. They are composed, among others, from *α*6*β*4 integrin, BP180, CD151, and plectin [21, 23]. Results of in vitro studies reveal that *α*6*β*4 integrin plays a key role in development of hemidesmosomes. This integrin is involved in connecting the intermediate filaments of a cell to the basement membrane and antibodies directed against this molecule inhibit both creation of hemidesmosomes and function of existing structures [23, 24]. Pemphigoid antigens BP180 and BP230 are linked not only to each other, but BP230 and *β*4 bind also with BP180 [21]. Interaction of BP180 and *α*6*β*4 integrin subunit is necessary for stabilization of hemidesmosomes structure [25, 26]. It was demonstrated that sole interaction between BP180 and plectin is not sufficient for hemidesmosome formation when lacking *α*6*β*4 integrin [21].

Complex interactions at the dermo-epidermal junction are also confirmed by other in vitro studies [27]. Studies in patients with ophthalmologic type of cicatrisans pemhigoid underline the role of antibodies directed against *β*4 integrin. Serum form patients with cicatrisans pemphigoid recognized a protein of molecular mass 205 kDa, for example, that of *β*4 integrin. Such protein was not recognized by sera from patients with bullous pemphigoid or pemhigus vulgaris [28–30].

Bhol et al. [31] revealed that serum from pemphigoid patients who had lesions on oral mucosa selectively binds human *α*6*β*4 integrin. In 48-hour culture with human oral mucosa obtained from the cheeks, this serum caused separation of epidermis from the basement membrane. Antibodies may bind intracellulary with specific domains leading in consequence to development of skin lesions [14, 31].

Leverkus et al. [27] using immunoblot method revealed decrease of antibodies' reactivity against BP180 and *β*4 integrin that correlated with decreased activity of ocular pemphigoid resulting from introduced treatment. Rashid et al. [32] examined sera from 20 patients with mucous membrane pemphigoid in active stage of the disease and demonstrated that all studies sera bound with *α*6 integrin subunit in immunoprecipitation and immunoblot methods. Focal loss of expression of *α*6*β*4 integrin is associated with loss of adhesion to the basement membrane.

Kurpakus et al. [33] examined the role of *α*6*β*4 in hemidesmosome formation on an in vitro model of wound healing and revealed that antibodies against *β*4 subunit did not disturb migration of epithelial cells but they initiated disruption of formerly assemblied hemidesmosomes. New hemidesmoses were also no longer formed and antibodies against *α*6*β*4 integrin completely isolated epithelial cells from the area of healing.

Our study revealed weak but linear and regular expression of *β*4 integrin in skin biopsies from healthy persons. Such expression proves that the presence of this molecule is constitutive in epidermal anchoring structures. However, in the course of immunologic delamination of the epidermis in BP, mediated by antibodies directed against basement membrane components and caused by a cascade of cytokine and enzymatic reactions, this expression was significantly changed. Irregular and focal expression of this integrin within the inflammatory infiltrates and blisters may reflect the destruction of the dermo-epidermal junction.

Selectins belong to the first family of adhesion molecules that initiate diapedesis, for example, rolling of leukocytes. This family encompasses three transporting glycoproteins with similar structure: leukocyte (L), platelet (P), and endothelial (E) selectin. Selectins are involved in leukocyte recruitment to the inflammatory foci and in the initial stages of inflammation play a role in rolling of leukocytes on the endothelium of blood vessels [18].

E-selectin is present in the stimulated endothelial cells. Production of this molecule is induced by a bacterial endotoxin, cytokines, thrombin, or tumor necrosis factor *α* (TNF*α*). Expression of E-selectin can be observed only in the foci of inflammation [18]. In our studies, expression of this selectin was observed in walls of blood vessels, slightly weaker in neutrophils. It confirms the role of the E-selectin in inflammatory process and development of pathological changes in pemphigoid.

D'Auria et al. [34] in a preliminary study examined serum concentration of soluble E-selectin in pemphigoid and pemphigus vulgaris patients and revealed its significant increase in patients in comparison with the control group, as well as significant correlation with a number of skin lesions. In course of therapy, both severity of skin lesions and concentration of studied selectin decreased in parallel, which may prove the usefulness of E-selectin as a treatment efficacy marker.

L-selectin is constitutively present on leukocyte surface and disappears after their stimulation. It has an important role in neutrophil recruitment to foci of inflammation and lymphocyte adhesion to endothelial cells of blood vessels in peripheral lymph nodes. The soluble form of this molecule is probably involved in regulation of adhesion processes [3, 18, 35]. Based on the fact that the inflammatory process is associated with leukocyte stimulation, lower expression of L-selectin in patients in comparison with the control group was expected in our study. Immunoexpression of L-selectin was however observed on lymphocytes, macrophages, and neutrophils. It may prove that not all inflammatory cells are simultaneously stimulated and some of them can still retain the L-selectin on the surface before its desquamation.

Recent literature data as well as results of our studies confirm the role of selectins and integrins in pathogenesis of pemphigoid. The elucidation of the role of inflammatory cells, their soluble mediators, adhesion molecules, and signal transduction pathways in the pathogenesis of the bullous diseases may be helpful in the development of new targeted therapies. Extensive research has focused on modulating activity of adhesive molecules. Among the new therapeutic modalities, humanized monoclonal antibodies against adhesion molecules are in early phase of clinical trials [36].

## Figures and Tables

**Table 1:** Mean immunoexpression of CD29, CD61, CD104, CD62E, and CD62L in BP and control group.

Adhesion molecule	CD29 (integrin *β*1)		CD61 (integrin *β*3)		CD104 (integrin *β*4)		CD62E (E-selectin)		CD62L (L-selectin)	
	Expression/patients		Expression/patients		Expression/patients		Expression/patients		Expression/patients	

Pemphigoid	+	14/21	+	14/21	+	14/21	+	15/21	+	15/21
	2+	4/21	2+	2/21	2+	4/21	2+	5/21	2+	2/21
	−	3/21	−	5/21	−	3/21	3+	1/21	−	4/21

Control group	+	3/10	+	0/10	+	8/10	+	5/10	+	4/10
	−	7/10	−	10/10	−	2/10	−	5/10	−	6/10
